# Robust and Reproducible Monoclonal Antibody Production Using a Transgenic Silkworm System

**DOI:** 10.3390/ijms262110287

**Published:** 2025-10-22

**Authors:** Seiki Yageta, Yudai Nagata, Mamoru Shimizu, Natsumi Yasaka, Takuma Iwasa, Takushi Nakajima, Takeo Kuwata, Shuzo Matsushita, Masahiro Tomita

**Affiliations:** 1 Immuno-Biological Laboratories, Co., Ltd., 1091-1 Naka Aza-Higashida, Fujioka 375-0005, Gunma, Japan; do-yageta@ibl-japan.co.jp (S.Y.); do-nagata@ibl-japan.co.jp (Y.N.); do-shimizu@ibl-japan.co.jp (M.S.); 2 CURED Inc., 8-16 Anseimachi Chuo-ku, Kumamoto 860-0801, Japan; nyasaka@cured-inc.com (N.Y.); t-iwasa@cured-inc.com (T.I.); tnakajima@cured-inc.com (T.N.); 3 The Joint Research Center for Human Retrovirus Infection, Kumamoto University, 2-2-1 Honjo, Chuo-ku, Kumamoto 860-0811, Japan; tkuwata@kumamoto-u.ac.jp (T.K.); shuzo@kumamoto-u.ac.jp (S.M.)

**Keywords:** transgenic silkworm, immunoglobulin G, recombinant protein production

## Abstract

Transgenic silkworms are promising host organisms for the production of therapeutic recombinant proteins due to their superior protein synthesis ability and human-like posttranslational modifications. In this study, we generated transgenic silkworms that secrete a recombinant human monoclonal antibody (mAb) against gp120 of human immunodeficiency virus (HIV) into their cocoons. Variations in the rearing temperature and humidity conditions had little effect on mAb properties, such as *N*-glycosylation. Next, we performed pilot-scale production of the mAb using three batches of transgenic silkworms. Rearing 22,000–45,000 silkworm larvae yielded 4–8 kg of cocoon shells per batch. Larval growth and development, as well as cocoon quality, were highly consistent across production batches. We extracted and purified the mAb from cocoon shells, yielding 6.1–7.6 g of purified mAb per kg of cocoons in each batch. Characterization of the purified mAb showed that the contents of oligomeric antibodies and host cocoon-derived proteins were less than 0.2% and 10 ppm, respectively, with high consistency among batches. From these results, we conclude that the transgenic silkworm system is sufficiently robust and reproducible for high-quality therapeutic mAb production.

## 1. Introduction

Technological advances for the mass production of high-quality and safe recombinant proteins have enabled their use as therapeutics for a wide range of diseases [1]. Cultured cells are primarily used as hosts to produce these therapeutic proteins. However, alternative recombinant protein production systems using transgenic animals or plants with superior recombinant protein synthesis capability are being developed to improve production efficiency and broaden the application of therapeutic proteins [2,3].

The silkworm *Bombyx mori* is a promising host organism for producing therapeutic proteins because its silk glands can synthesize large amounts of silk proteins. Recombinant genetic technologies have been applied to generate transgenic silkworms [4] that can synthesize large amounts of recombinant proteins in their silk glands [5,6]. This system has several advantages for recombinant protein production. As the foreign gene encoding the target recombinant protein is inserted into the silkworm genome using a *piggyBac* transposon-based vector, the silkworm offspring can inherit the inserted genes. As a silkworm moth lays 300–500 eggs, the scale of production can be expanded easily by increasing the number of mated moths. The silk glands consist of the anterior and posterior silk glands, both capable of expressing recombinant proteins. Expression in the posterior silk glands allows proteins to be localized within the core fibroin fibers [5,7,8], potentially enabling higher protein production. On the other hand, expression in the middle silk glands leads to the localization of proteins within the sericin layer surrounding the fibroin fibers, offering the advantage of easier protein extraction [6,9]. The middle silk glands expression system has been applied successfully for the production of recombinant mammalian proteins with complex structures, such as glycoproteins and multisubunit proteins, including monoclonal antibodies (mAbs) [10,11], interferon [12], and fibrinogen [13].

Iizuka et al. [10] first generated transgenic silkworms expressing recombinant mouse mAb in their cocoons. The mAb extracted from the cocoons was a tetramer consisting of two heavy chains and two light chains, which showed identical antigen binding properties to the original mAb produced by a hybridoma. Tada et al. [11] also established transgenic silkworms expressing a human–mouse chimeric anti-CD20 mAb and performed more detailed characterization. While the silkworm-derived mAb exhibited similar antigen-binding properties to that produced by Chinese hamster ovary (CHO) cells, differences in the effector functions were observed, including stronger antibody-dependent cell-mediated cytotoxicity (ADCC) and weaker complement-dependent cytotoxicity. Analysis of posttranslational modifications revealed major differences in *N*-glycan structures between the silkworm- and CHO-derived mAbs, but no differences in other modifications. The unique effector functions of the mAb produced in transgenic silkworms were considered to be attributable to its characteristic *N*-glycan structures, i.e., the lack of core fucose and galactose at the nonreducing terminus.

To ensure the therapeutic application of mAbs produced by transgenic silkworms, it is necessary to verify that this system can consistently produce high-quality products that meet the required specifications for purity, potency, safety, efficacy, stability, and other quality attributes [14]. In the present study, we generated transgenic silkworms expressing an anti-human HIV gp120 V3 loop human mAb [15], and analyzed several molecular properties of the silkworm-derived mAb, including antigen binding properties, ADCC, *N*-glycan structure, and glycosylation state, i.e., the distribution of hemi- and nonglycosylated forms. Then, we investigated the impacts of rearing conditions, particularly temperature and/or humidity during the fourth- to fifth-instar and cocooning stages, on the properties of the mAb. Finally, we conducted three batches of pilot-scale production of the mAb, starting with 22,000–45,000 first-instar larvae and producing 4–8 kg of mAb-containing cocoon shells per batch. Subsequent purification from 4–8 kg of cocoons yielded 20–60 g of mAb per batch. The contents of oligomeric antibodies and host cocoon proteins (HCPs) were less than 0.2% and 10 ppm, respectively, indicating the high quality of the silkworm-derived mAb.

## 2. Results

### 2.1. Generation of mAb-Expressing Transgenic Silkworms

To create transgenic silkworms expressing anti-HIV gp120 V3 loop recombinant human mAb (1C10) in their cocoons, the 1C10/pMSG3.1MG vector containing the cDNAs of 1C10 heavy and light chains was constructed. This vector was then injected into the eggs of silkworm strain *w1-pnd* along with the helper vector pHA3PIG. The transgenic silkworms were screened from G1 embryos, which were obtained by mating G0 moths, based on Monster Green Fluorescent Protein (MGFP; Promega, Madison, WI, USA) expression in the eyes. These transgenic silkworms were sibling-mated to generate a homozygous transgenic silkworm line (1C10W^+/+^). The experimental silkworm strain *w1-pnd* produces small cocoons (shell weight: 60–70 mg). Therefore, the *w1-pnd*-based 1C10W^+/+^ line was repeatedly crossed with Kinshu, a practical strain that produces large cocoons, followed by sibling mating to establish a 1C10-homozygous line. The resulting 1C10K^+/+^ line was crossed with a homozygous IM71K^+/+^ line carrying the baculovirus *trans*-activator *ie1* gene, generating a Kinshu-based 1C10K^+/−^/IM71K^+/−^ line. This line produced cocoons with an average cocoon shell weight of 243 mg.

### 2.2. Characterization of the Silkworm-Derived mAb

The silkworm-derived mAb was extracted and purified from cocoons of the 1C10K^+/−^/IM71K^+/−^ line. The mAb produced by this line was designated as 1C10K. We also prepared purified mAb (1C10C) produced by CHO cells. Both mAbs were then characterized as outlined below.

**Figure 1:**
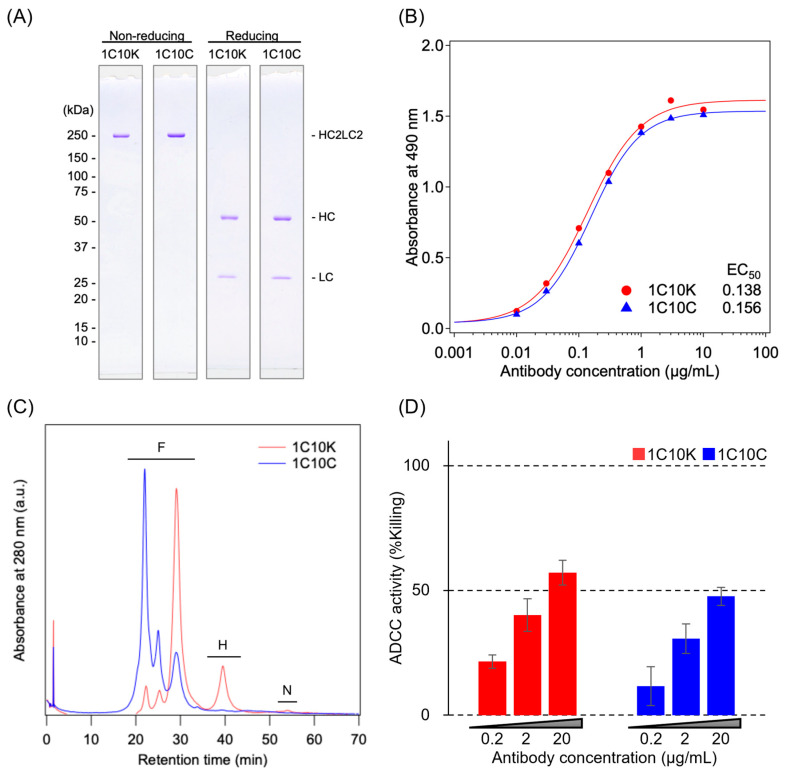
Characterization of purified 1C10K and 1C10C. (**A**) Sodium dodecyl sulfate polyacrylamide gel electrophoresis (SDS-PAGE). Disulfide bonds of monoclonal antibodies (mAbs) were left intact (non-reducing conditions) or reduced by 0.1 mM dithiothreitol (reducing conditions). Molecular weights (kDa) determined using protein standards are indicated on the left. On the right, HC2LC2, HC, and LC indicate a tetramer of two heavy chains and two light chains of IgG, a heavy-chain monomer, and a light-chain monomer, respectively. (**B**) Antigen-binding properties of 1C10K (red circles) and 1C10C (blue squares) determined by enzyme-linked immunosorbent assay (ELISA). Serially diluted mAbs were reacted with a fixed amount of immobilized antigen. Half-maximal effective concentration (EC_50_) values were determined using a four-parameter logistic model. (**C**) *N*-glycosylation status of mAbs analyzed by cation-exchange chromatography (CEX). Each chromatogram was obtained with a 0–1 M sodium chloride gradient. F, H, and N indicate fully glycosylated, hemiglycosylated, and nonglycosylated immunoglobulin G (IgG), respectively. (**D**) Antibody-dependent cell-mediated cytotoxicity (ADCC) assay using NKR24 cells infected with human immunodeficiency virus (HIV)-1 BaL and N6 effector cells. ADCC was evaluated based on the cell death rate, calculated from relative light units (RLU), when the mAbs were added at concentrations of 0.2, 2, and 20 μg/mL.

1C10K and 1C10C were analyzed by sodium dodecyl sulfate–polyacrylamide gel electrophoresis (SDS-PAGE) (Figure 1A). Under reducing conditions, both mAbs showed two bands, with molecular weights of 50 and 25 kDa, corresponding to the mAb heavy and light chains, respectively. There was no marked difference between the two mAbs. Under nonreducing conditions, a single band corresponding to the intact mAb (a complex of two heavy chains and two light chains) was observed for both mAbs. The apparent molecular weight of this band was approximately 250 kDa, which differed from the calculated value of 150 kDa based on the amino acid sequence. Such mobility shifts are often observed on SDS-PAGE results for nonreduced mAbs [16].

The binding of 1C10K and 1C10C to the immobilized antigen (HIV gp120 V3 loop peptide) was analyzed by enzyme-linked immunosorbent assay (ELISA) (Figure 1B). The binding curves were nearly identical, with similar half-maximal effective concentration (EC_50_) values, indicating that both mAbs had similar antigen binding properties.

The *N*-linked glycosylation statuses of 1C10K and 1C10C were analyzed by cation-exchange chromatography (CEX) (Figure 1C). Although immunoglobulin G (IgG) generally bears an *N*-linked glycosylation site at Asn297 on each of two heavy chains, a previous study showed that silkworm-derived mAbs include hemi- and nonglycosylated forms [17]. In the present study, CEX analysis of 1C10K revealed peaks corresponding to the fully, hemi-, and nonglycosylated forms. The fully glycosylated form consisted of three peaks, with the major peaks differing between 1C10K and 1C10C. Although the precise mechanisms remain unclear, this result may have been due to differences in posttranslational modifications that contribute to charge heterogeneity, including N-terminal pyroglutamate, succinimide intermediate, and deamidation [18]. The peak area ratios of the hemi- and nonglycosylated forms of 1C10K were 15.9% and 3.6%, respectively. In 1C10C, the amounts of these forms were below the limits of detection.

**Figure 2:**
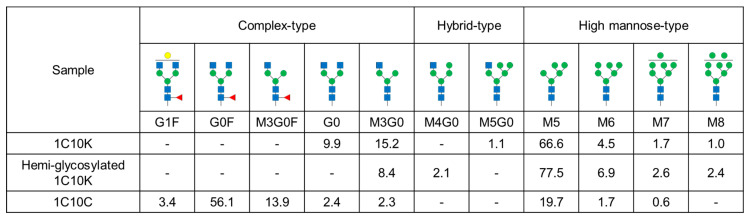
Percentage distribution of *N*-glycan structures. Blue squares indicate *N*-acetyl-d-glucosamine (GlcNAc); green circles indicate d-mannose; yellow circles indicate d-galactose; red triangles indicate l-fucose.

The *N*-glycan structures of 1C10K and 1C10C were analyzed by matrix-assisted laser desorption/ionization time-of-flight mass spectrometry (MALDI-TOF MS). The percentages of detected glycan structures are shown in Figure 2. In 1C10K, high-mannose-type glycans accounted for 73.8% of the total, while complex-type glycans accounted for 25.1%. In the complex-type glycans, neither core fucosylation nor galactosylation was detected. In contrast, the majority of glycans in 1C10C were complex-type (78.1%), most of which had core fucosylation. The high-mannose-type glycans accounted for 22.0%. We further analyzed the glycan structures of the hemiglycosylated form of 1C10K after fractionation by CEX. The distribution of glycan structures was similar to that of 1C10K before separation, but the percentage of high-mannose-type glycans in the hemiglycosylated form (89.4%) was higher than in the unseparated 1C10K (73.8%).

The ADCC of 1C10K and 1C10C was analyzed using CEM.NKr-CCR5 cells with long-terminal repeat-luciferase (LTR-Luc) infected with HIV-1 BaL as target cells, and N6 cells as effector cells (Figure 1D). ADCC was higher for 1C10K than for 1C10C when mAbs were added at concentrations ranging from 0.2 to 20 μg/mL.

### 2.3. Effects of Rearing Conditions on Silkworm Rearing Outcomes and 1C10K Quality

Rearing temperature and humidity were considered critical process parameters likely to significantly impact the quality of 1C10K in cocoons. First, we examined the effects of rearing temperature during the fourth- and fifth-instar stages. Each of the 20 silkworms was reared from the early fourth-instar stage to the late fifth-instar stage at five different temperatures (20 °C, 23 °C, 25 °C, 27 °C, and 30 °C), and subsequent cocooning was conducted at 25 °C. As shown in Table 1, changes in rearing temperature significantly affected rearing outcomes (i.e., rearing period, cocoon formation rate, and cocoon shell weight). The silkworms took 11 days to grow from the first day of the fourth-instar stage to the end of the fifth-instar stage at 25 °C, 27 °C, and 30 °C. However, this period was extended to 17 and 13 days for silkworms reared at 20 °C and 23 °C, respectively. While all larvae reared at 25 °C, 27 °C, and 30 °C successfully formed cocoons, the cocoon formation rates declined to 90% and 95% at 20 °C and 23 °C, respectively. The cocoon shell weight was approximately 250 mg for larvae reared at 23 °C, 25 °C, and 27 °C, but decreased slightly when reared at 20 °C and 30 °C. The 1C10K in cocoons produced at each temperature was purified and its *N*-glycan status and structure were analyzed. There were no significant differences in the rates of hemi- and nonglycosylated forms among silkworms reared at different temperatures. Similarly, the *N*-glycan structures of 1C10K showed little variation across temperatures, although the proportion of high-mannose-type *N*-glycans was slightly lower at 20 °C and 23 °C than at other temperatures.

Next, we investigated the effects of temperature and humidity on the cocooning process. Seven sets of conditions were prepared with different temperatures (20–30 °C) and relative humidity (RH, 25–60%) settings (Table 2), and 20 silkworms were reared under each condition from the late fifth-instar stage until completion of cocoon formation. Temperature affected the cocooning period and cocoon shell weight, independent of the humidity level. Silkworms finished cocooning within 8 days at 25 °C and 30 °C, but within 10 days at 20 °C. The cocoon shell weight was highest at 25 °C, followed by 20 °C and 30 °C. Both humidity and temperature affected the extraction efficiency of 1C10K from the cocoons. In particular, high RH had a significant impact on extraction efficiency. For example, when compared within a temperature of 25 °C, the extraction rate at 25% RH was 80%, while at 45% RH and 65% RH, the extraction rates dropped to 65% and 58%, respectively. High temperatures under high RH conditions also significantly decreased the extraction efficiency. At 65% RH, the extraction rate was 70% at 20 °C, but dropped to 53% at 30 °C. Next, we characterized the purified 1C10K from cocoons under each condition. SDS-PAGE, size exclusion chromatography (SEC), and antigen-immobilized ELISA revealed no significant differences in the characteristics of 1C10K produced under each condition.

From these results, we concluded that changes in temperature during the fourth- and fifth-instar larval stages and those in temperature and RH during the cocooning stage affect silkworm rearing and cocooning outcomes, but have little effect on the quality of 1C10K. Based on the rearing and cocooning performance, the optimal temperature for the fourth- and fifth-instar larval stages was determined to be 25 °C, while the optimal temperature and RH for the cocooning stages were 25 °C and 25% RH, respectively.

### 2.4. Pilot-Scale Silkworm Rearing

We performed three pilot-scale batches of silkworm rearing to confirm that our process could repeatedly produce 1C10K-containing cocoons with constant yield and quality. The eggs of the 1C10K^+/−^/IM71K^+/−^ line used for each batch were prepared independently by mating 1C10K^+/+^ and IM71K^+/+^ moths from different generations. Batches A, B, and C were started with 28,000, 45,000, and 22,000 first-instar larvae, respectively. The rearing temperature for larvae was set at 25 °C, and the cocooning temperature and RH were set at 25 °C and 25%, respectively.

**Figure 3:**
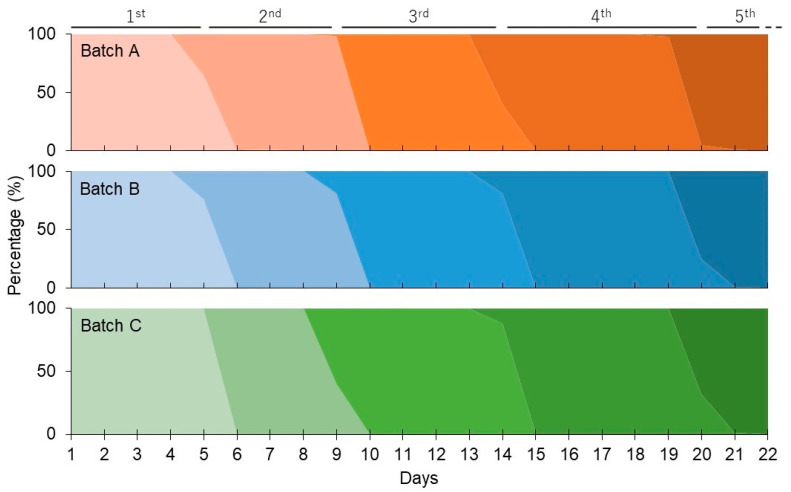
Larval instar transition. The percentages of larvae at each instar stage (from first to fifth) on each rearing day are shown. Batches A, B, and C are represented by red, blue, and green, respectively. Darker shading indicates later instar stages, with the corresponding stage indicated above.

Larval growth and development were analyzed by measuring the transition of larval instars, survival rate, and body weight. Silkworm larvae typically undergo four molts to reach the fifth-instar stage. As shown in Figure 3, the larvae in the present study also developed through the fifth-instar stage, with four short molting periods between each instar stage. The four molts in the three batches began on nearly the same days after the start of rearing and ended within 1–2 days, indicating that the larvae grew synchronously within each batch and that growth was comparable among batches. The survival rate and body weight of each instar stage in the three rearing batches are shown in Table 3. Although survival rates decreased gradually from the third- to fifth-instar stage, the reduction was similar among the three batches. More than 80% of larvae survived in the fifth-instar stage in all three batches. The average larval body weights from the fourth- to late fifth-instar stage were similar across the three batches. The small standard deviations in body weight indicated minimal individual variation in larval growth.

As shown in Table 3, batches A, B, and C produced 4.6, 8.0, and 4.0 kg of cocoon shells, respectively. The average weights of single cocoon shells were 222, 226, and 221 mg, respectively. A portion of cocoon shells from each batch was sampled, and 1C10K was extracted from these and quantified using Protein A affinity chromatography, as described in Materials and Methods. The 1C10K concentrations were nearly identical among extracts from different batches (0.11, 0.12, and 0.11 mg/mL for batches A, B, and C, respectively).

### 2.5. Purification of 1C10K from Cocoons

The 1C10K was extracted and purified from each batch of cocoons. First, 1C10K was extracted by immersing the cocoons in extraction buffer (20 mM sodium phosphate, pH 7.5, 150 mM sodium chloride, 4 M urea, and 0.2% *w*/*v* Triton X-100). In this process, the pH 7.5 buffer was used for the extraction of 1C10K. Although its composition differed from that of the pH 5.3 buffer used in the small-scale purification described in Section 2.2, there was no significant difference in the amount of 1C10K and impurity proteins extracted. Then, protein aggregates and other insoluble impurities in the extract were removed by depth filtration. Subsequently, 1C10K was purified in three chromatography steps using Protein A affinity, hydroxyapatite, and multimodal strong anion exchange resins. Finally, 1C10K was concentrated and the buffer solution was exchanged by tangential flow filtration (TFF). For each of the three purification batches, the process solutions were sampled at each purification step. The levels of 1C10K, HCPs and oligomeric IgGs were analyzed to evaluate the yield, impurity reduction efficiency, and process reproducibility.

**Figure 4:**
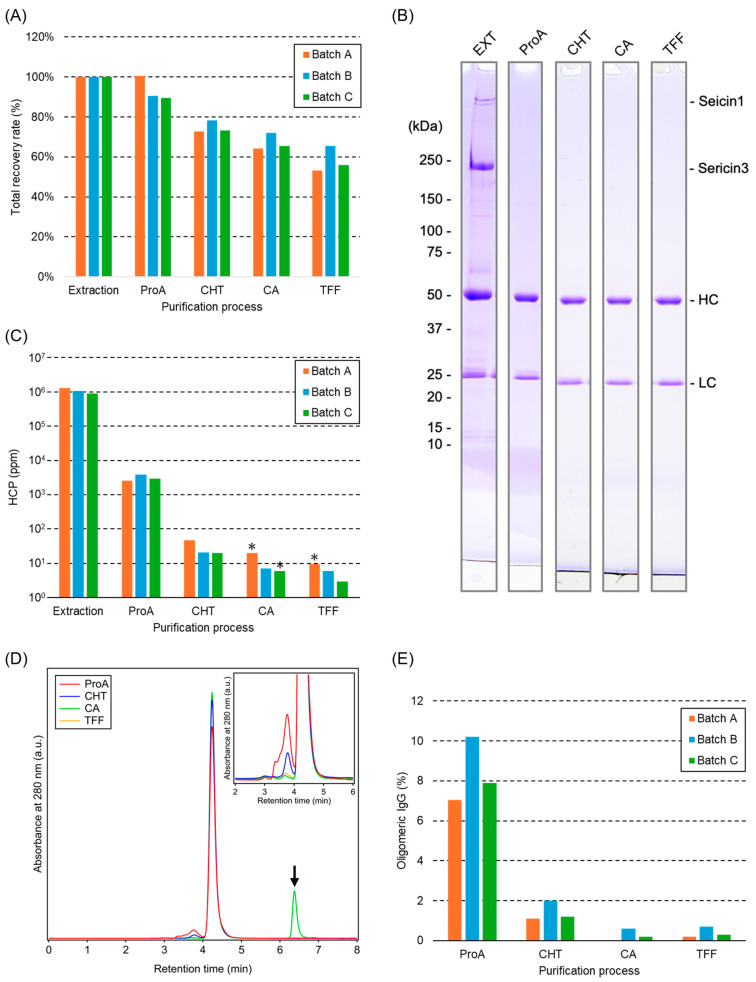
Process analyses of three batches of pilot-scale purification involving extraction (EXT), Protein A chromatography (ProA), ceramic hydroxyapatite chromatography (CHT), multimodal anion exchange chromatography (CA), and tangential flow filtration (TFF). (**A**) Trends in the recovery rates of 1C10K for each purification process. (**B**) SDS-PAGE under reducing conditions for each purification step in batch B. HC and LC indicate heavy and light chains of IgG, respectively. (**C**) Residual host cocoon proteins (HCP) levels, measured by an HCP assay. Asterisks indicate values below the lower limit of quantitation, and in such cases, the height of the bars represents those limits. (**D**) Size exclusion chromatography (SEC) chromatograms of each purification step in batch B. Black arrow indicates citric acid, a component of the sample solution. Inset shows a detailed view of elution peaks of the oligomeric IgGs. (**E**) Ratio of residual oligomeric IgGs measured by SEC.

Figure 4A shows the recovery rates of 1C10K in each purification step. The recovery rate in Protein A chromatography was slightly lower in batches B and C than in batch A, but there were no other differences among batches. SDS-PAGE of process solutions from batch B revealed that major protein impurities, including sericin 1 and 3, were effectively removed after Protein A column chromatography (Figure 4B). The same results were obtained for batches A and C. The levels of HCPs at each purification step were quantified by ELISA, and no significant differences were detected among the three batches (Figure 4C). The eluted fraction from the Protein A column contained HCPs at levels of 2547–3791 ppm. After the TFF step, the levels were reduced to less than 9.5 ppm. Oligomeric IgGs were analyzed by SEC. As an example, the SEC chromatogram of batch B is shown in Figure 4D. The main peak corresponded to monomeric IgGs, while oligomeric IgGs appeared as smaller peaks at earlier retention times. Figure 4E presents the proportion of oligomeric IgGs at each purification step in the three batches, indicating consistent results among batches. The percentages of oligomeric IgGs in the Protein A eluate were 7.1–10.2% but decreased to 0.2–0.7% after TFF.

The yields and recoveries of purified 1C10K are summarized in Table 4. As starting material for purification, 4 kg of cocoon shells were used for batches A and C, and 8 kg for batch B. The total amounts of 1C10K in the extracts were 40.6, 88.2, and 43.7 g for batches A, B, and C, respectively. Therefore, the amounts of 1C10K extracted per kilogram of cocoons were 10.2, 11.0, and 10.9 g, respectively, showing similar extraction efficiency among the three batches. After all purification steps, 21.6, 57.8, and 24.4 g of purified 1C10K were obtained from batches A, B, and C, respectively. The overall recovery rates of the purified 1C10K relative to the amounts in the extracts were 53%, 66%, and 56% for batches A, B, and C, respectively.

### 2.6. Quality of Purified 1C10K

To confirm consistent quality of purified 1C10K among the three batches, we evaluated antigen-binding activity, *N*-glycosylation status, *N*-glycan structure, oligomeric IgGs, and HCPs (Table 5).

Antigen-binding activity was nearly identical among batches A, B, and C, with similar EC_50_ values of 0.61, 0.54, and 0.54 μg/mL, respectively. The percentages of hemiglycosylated IgG were 16.7%, 16.2%, and 16.7% in batches A, B, and C, respectively, while those of nonglycosylated IgG were 0.5%, 0.4%, and 0.6%, respectively. *N*-glycan profiling revealed that the percentages of high-mannose-type glycans were 71.6%, 71.0%, and 72.5%, and those of complex-type glycans were 28.4%, 30.1%, and 27.5%, respectively. No core fucosylation was detected in any of the batches. Oligomeric IgGs were detected at 0.2% in all batches. HCP levels were 3.9, 4.6, and 2.9 ppm in batches A, B, and C, respectively. These results indicate excellent batch-to-batch consistency.

## 3. Discussion

We generated transgenic silkworms expressing 1C10K mAb. A comparison between 1C10K and the CHO cell-derived 1C10C revealed identical antigen binding activity but differences in glycosylation status and glycan structures. The hemi- and nonglycosylated forms were present at nonnegligible levels in 1C10K but were barely detectable in 1C10C. Although such aberrant forms were observed previously in silkworm- and *Pichia*-derived mAbs [17,19], the mechanism underlying their formation remains unclear. There may be insufficient amounts of oligosaccharyltransferases in the endoplasmic reticulum of silk gland cells, or the functions of silkworm oligosaccharyltransferase subunits, such as substrate recognition, may differ from those of their mammalian counterparts [20,21]. Regardless, it remains necessary to investigate the stability, immunogenicity, and other properties of the hemi- and nonglycosylated forms of 1C10K.

The *N*-glycan structures of 1C10K were mainly of the high-mannose type, with small amounts of complex-type glycans also detected, and core fucosylation was not observed. These observations were consistent with those of a previous study by Iizuka et al. [10]. Among the high-mannose-type and complex-type glycans, the most abundant structures were M5 and M3G0. The *N*-glycan processing reaction from M5 to G0 proceeds sequentially through the *N*-glycan processing enzymes, i.e., glucose *N*-acetyltransferase I (GnT-I), α-mannosidase II, and glucose *N*-acetyltransferase II [22,23]. In addition, Kajiura et al. [24] recently reported that BmFDL, a β-*N*-acetylglucosaminidase, cleaves GlcNAc bound to the nonreducing terminal α1,3-linked mannose residue in the middle silk gland of the silkworm. Therefore, the GlcNAc addition reaction catalyzed by GnT-I may represent a rate-limiting step, leading to the accumulation of M5. The accumulation of M3G0 instead of G0 may be due to the trimming reaction of the GlcNAc residue from G0 by BmFDL. We found that 1C10K exhibited higher ADCC than 1C10C. Ha et al. [19] reported that the hemiglycosylated form significantly reduced binding affinity to FcγRIIIA, decreasing ADCC by 3.5-fold. However, afucosylated glycans, including high-mannose-type glycans, have been reported to enhance ADCC by increasing binding affinity to FcγRIIIA [25]. The higher ADCC of 1C10K suggests that the positive effect of the absence of fucosylated glycans outweighed the negative effect of the hemiglycosylated form.

One of the concerns with insect-type *N*-glycans is their potential immunogenicity, especially the presence of α-1,3-fucose, which is known to be highly immunogenic [26]. However, as noted above, no core-fucosylated glycans, including those containing α-1,3-fucose, were detected in 1C10K. This result is consistent with previous studies reporting mAb expression in the middle silk gland of silkworms [10,11,17]. Furthermore, a recent study reported the administration of recombinant α-L-isorhamnidase expressed in silkworm silk glands to Japanese macaques [27]. In this study, the silkworm-derived enzyme was administered intravenously at a dose of 0.58 mg/kg body weight. No serious adverse effects, including anaphylactic shock, were observed over a trial period of more than 400 days. While the immunogenicity of 1C10K will need to be investigated in the future, it might not represent a major concern.

We investigated the effects of rearing temperature during the fourth- and fifth-instar stages on rearing outcomes of silkworms and the quality of 1C10K in cocoons. During these stages, the middle silk glands develop rapidly and large amounts of 1C10K are synthesized because transcription of 1C10K is designed to be driven by the promoter of *sericin1*, an endogenous silk protein gene [6]. Lower temperatures resulted in longer rearing periods and lower cocoon formation rates compared to the optimal rearing temperature of 25 °C. Additionally, cocoon shell weight decreased at both higher and lower temperatures. In contrast, the percentages of hemi- and nonglycosylated forms, and the distribution of *N*-glycan structures did not change significantly with variation in rearing temperature. As ADCC is an important property for treating HIV-related diseases, the distributions of *N*-glycosylation status and *N*-glycan structures are considered critical quality attributes that must be strictly controlled by appropriate process parameters. Therefore, robustness against variations in rearing temperature would be advantageous for maintaining consistent drug efficacy. The effects of temperature and RH during the cocooning stage on 1C10K production were also examined. At this stage, 1C10K synthesis in the middle silk glands is almost completed, and 1C10K is secreted into the cocoons together with endogenous silk proteins. As a result, higher RH reduced the extraction efficiency of 1C10K from cocoons. During the silk reeling process in the sericulture industry, high humidity during cocooning decreases the reelability of silk threads from cocoons [28]. Under high-RH conditions, the higher-order structures of sericin proteins, which hold the two fibroin filaments together like glue [29], change from an amorphous state to a crystalline state due to moisture absorption, making sericin difficult to dissolve [30]. Therefore, it is likely that in cocoons produced under high-RH conditions, 1C10K is surrounded by the insoluble sericin meshwork, making it difficult to extract. Thus, high humidity during the cocooning stage affects the yield of 1C10K. However, we have shown that high humidity does not affect the quality of 1C10K. These results confirm that deviations from the optimal temperature and humidity affect the yield of 1C10K but have little effect on its quality. The transgenic silkworm system proved to be a robust platform to produce 1C10K with consistent quality.

In the present study, the pilot-scale rearing of transgenic silkworms followed by purification was conducted in three independent batches to evaluate the reproducibility of 1C10K production. All batches exhibited similar larval growth patterns, and showed comparable survival rates and cocoon yields. The concentrations of 1C10K in cocoons, extraction efficiencies from cocoons, and recovery rates during purification were consistent across batches. Furthermore, the purified antibodies exhibited excellent batch-to-batch consistency in terms of antigen binding activity, ratio of hemi- and nonglycosylated forms, and *N*-glycosylation profiles. In all batches, oligomeric IgGs and HCPs were maintained below the conventionally acceptable levels of 5% and 100 ppm, respectively [31].

As noted above, our transgenic silkworm system minimized the impact of variations in the rearing environment and scale, ranging from small-scale (20 silkworms) to pilot-scale (tens of thousands), on mAb quality. Furthermore, the pilot-scale production of three batches demonstrated excellent reproducibility in terms of mAb recovery and quality, which were achieved by controlling only a few process parameters: diet amount, temperature, and humidity. In contrast, in protein production using mammalian cell culture systems, such as those based on CHO cells, many process parameters, including cell density, agitation speed, temperature, pH, osmolality, dissolved O_2_ and CO_2_ levels, as well as nutrient and metabolite concentrations, significantly affect product quality, including *N*-glycan structures [32]. Therefore, consistent product quality cannot be maintained unless these parameters are tightly controlled. It is also well known that differences in production sites for cell culture affect product quality [33]. The silkworm, which we used as a host for protein production, regulates gas exchange, nutrient supply, and metabolite excretion at the individual level through its hemolymphatic and tracheal respiratory systems. Thus, a single silkworm can be regarded as a small bioreactor capable of autonomously regulating process parameters. These intrinsic features of the silkworm as a host organism may account for the robustness, reproducibility, and scalability of mAb production demonstrated in this study.

In conclusion, the transgenic silkworm system can be considered an ideal platform for the production of therapeutic proteins that require consistent quality. Furthermore, silkworm rearing techniques developed through the sericultural industry may enable the large-scale production required for the practical application of mAb drugs. In contrast, the transgenic silkworm system currently exhibits insufficient productivity and lacks a cost advantage compared to the CHO cell system. We believe that this platform can be made more economically viable not only by increasing protein expression levels, but also by developing related technologies such as mechanizing the rearing process and creating low-cost artificial diet.

## 4. Materials and Methods

### 4.1. Animals

The silkworm *B. mori w1-pnd* strain was obtained from the National Agriculture and Food Research Organization (Tsukuba, Japan). The Kinshu strain was purchased from Uedasanshu Co., Ltd. (Nagano, Japan). The larvae were reared at 25 °C on an artificial diet (Silk Mate PM; Nosan, Kanagawa, Japan). A cool incubator (CN-40A; Mitsubishi Electric Engineering Co., Ltd., Tokyo, Japan), equipped with a humidity control unit (AHCU-2; Kitz Microfilter, Nagano, Japan), was used for rearing under various temperature and humidity conditions.

### 4.2. Vector Construction

The cDNAs encoding the full-length heavy and light chain open reading frames (ORFs) of a human mAb against the gp120 V3 loop of HIV (1C10) were amplified by polymerase chain reaction from the vectors pMPE-1C10 and pKVA2-1C10, respectively [15]. The 5′-UTR sequence of the *B. mori* nucleopolyhedrovirus polyhedrin gene was inserted upstream of the cDNAs of the heavy and light chain ORFs. The cDNA for the heavy chain with the 5′-UTR was inserted into the *Nru*I site located downstream of the *sericin1* promoter in pMSG3.1MG [17], and the light-chain cDNA with the 5′-UTR was inserted into the *Eco*47III site located downstream of another *sericin1* promoter within the vector, generating 1C10/pMSG3.1MG.

### 4.3. Generation of Transgenic Silkworms

The constructed vector 1C10/pMSG3.1MG and helper vector pHA3PIG were injected into eggs of the silkworm *w1-pnd* strain [4]. From G1 embryos obtained by mating of G0 moths, transgenic silkworms were screened for MGFP expression in the eyes. The copy numbers of the inserted 1C10 cDNAs were analyzed by Southern blotting using the genomic DNA extracted from MGFP-positive G1 moths to select G1 transgenic lines with a single copy of the inserted DNAs. The selected lines were sibling-mated, and a homozygous transgenic silkworm line (1C10W^+/+^) was obtained from the G2 generation. The 1C10W^+/+^ line was crossed with the Kinshu silkworm strain, followed by continuous back-crossing with Kinshu six times to establish a line with a homogenous genetic background (1C10K^+/−^). Finally, a Kinshu-based homozygous transgenic line (1C10K^+/+^) was established by sibling mating. To express 1C10K mAb, the 1C10K^+/+^ line was crossed with a Kinshu-based IM71 silkworm line (IM71K^+/+^) carrying the baculovirus *trans*-activator *ie1* gene [17], generating a silkworm line (1C10K^+/−^/IM71K^+/−^) bearing both the 1C10 cDNAs and *ie1* gene.

### 4.4. Extraction and Purification of 1C10K mAb from Silkworm Cocoons

The 1C10K was extracted from the cocoons by stirring in extraction buffer (60 mM sodium acetate, pH 5.3, 30 mM sodium chloride, 4 M urea, and 0.2% *w*/*v* Triton X-100) for 1 h. After the extract was collected, the cocoons were rinsed with extraction buffer, and the rinse was added to the initial extracts. The extract was clarified by filtration using a 10-μm stainless steel filter unit (SMC, Tokyo, Japan). The 1C10K in the extract was purified by two-step liquid chromatography consisting of cation exchange chromatography and Protein A affinity chromatography. Briefly, the filtered extract was loaded onto a StreamLine cation exchange chromatography column with 5 L of StreamLine SP resin (Cytiva, Marlborough, MA, USA) that had been equilibrated and expanded by upward liquid flow with extraction buffer. The flow rate was set to 1.5 L/min. The 1C10K-bound resin was washed with wash buffer (60 mM sodium acetate, pH 5.3, and 30 mM sodium chloride), and the flow was then stopped to allow the resin to settle. The bound 1C10K was eluted with elution buffer (60 mM sodium acetate, pH 5.1, and 300 mM sodium chloride) at a flow rate of 0.5 L/min. The 1C10K solution was concentrated and the buffer was exchanged with phosphate-buffered saline (PBS) by TFF using a Pellicon 2 Mini TFF system with a Biomax 100 kDa membrane (Merck, Darmstadt, Germany). The solution was then applied to a Protein A column packed with 1 L of MabSelect SuRe resin (Cytiva), which had been pre-equilibrated with PBS. The flow rate was set to 176 mL/min. After the column was washed with PBS, the bound 1C10K was eluted with elution buffer (100 mM sodium citrate, pH 3.0). Then, the pH was neutralized to 7.4 by adding 1 M tris solution. The volume of 1C10K solution was concentrated, and the buffer was exchanged with TFF buffer (10 mM sodium acetate, pH 5.5, 50 mM sodium chloride, 100 mM arginine) using the TFF system as described above. The concentration of 1C10K was adjusted to 20 mg/mL, and the resultant solution was used in analyses.

### 4.5. Determination of the Extraction Efficiency of 1C10K from Cocoons

Total proteins contained in the sericin layer of silk fibers were extracted by treating the cocoons with 8 M urea, 2 vol% β-mercaptoethanol, and 50 mM tris-HCl (pH 8.0) at 80 °C for 5 min. Triton-extracted proteins were also prepared using extraction buffer (60 mM sodium acetate, pH 5.3, 30 mM sodium chloride, 4 M urea, and 0.2% *w*/*v* Triton X-100), as described in Section 4.4. Total proteins and Triton-extracted proteins were subjected to SDS-PAGE under reducing conditions as described in Section 4.6. The extraction efficiency of 1C10K was calculated as the ratio of the band density of 1C10K heavy chains in the Triton-extracted proteins to that in total proteins.

### 4.6. SDS-PAGE

Protein samples were mixed with 5× SDS-PAGE sample buffer (0.5 M tris-HCl, pH 6.8, 10% *w*/*v* SDS, 50% *w*/*v* glycerol, and 0.03% *w*/*v* bromophenol blue). For electrophoresis under reducing conditions, dithiothreitol was added to the sample at a final concentration of 100 mM. The prepared samples were incubated at 95 °C for 5 min and applied to wells of a 5–20% polyacrylamide gradient gel (e-PAGEL; ATTO, Tokyo, Japan). Precision Plus Protein Dual Color Standards (Bio-Rad Laboratories, Hercules, CA, USA) were used as a protein standard. Electrophoresis was performed at 20 mA per polyacrylamide gel for 80 min with running buffer (25 mM tris, 0.1% *w*/*v* SDS, and 193 mM glycine). The gels were stained with staining solution (50 vol% methanol, 10 vol% acetic acid, and 0.2% *w*/*v* Coomassie brilliant blue R-250), and then destained with destaining solution (20 vol% methanol and 10 vol% acetic acid) at room temperature. Gel images were captured using a scanner (ES-2200; Seiko Epson, Nagano, Japan). Band intensity levels were analyzed using ImageJ software version 1.47t [34].

### 4.7. ELISA

The reactivity of mAbs against a V3 epitope was analyzed by peptide-based ELISA as described previously [15], with slight modifications. Briefly, 50 ng of synthetic V3 peptides in 50 μL of 0.1 M sodium carbonate (pH 9.5) was added to MaxiSorp 96-well plates (Thermo Fisher Scientific, Waltham, MA, USA), followed by incubation at 4 °C for 16 h to coat the wells. The plates were then blocked with 200 μL of 0.1% bovine serum albumin in PBS at 4 °C for 16 h. Diluted mAbs (50 μL) were added to the wells, and the plates were incubated at room temperature for 1 h. The mAbs bound to V3 peptides were detected with 50 μL of horseradish peroxidase (HRP)-conjugated Goat anti-human IgG (Southern Biotech, Birmingham, AL, USA), followed by the addition of HRP substrate, 0.4 mg/mL *o*-phenylenediamine dihydrochloride (Merck) in 100 μL of 50 mM citrate-phosphate buffer (pH 5.0) containing 0.03 vol% H_2_O_2_. Absorbance at 490 nm was measured using a SpectraMAX ABS microplate reader (Molecular Devices, San Jose, CA, USA). EC_50_ values were calculated by fitting the data to a four-parameter logistic model using SoftMax Pro software version 7.1 (Molecular Devices).

### 4.8. High-Performance Liquid Chromatography (HPLC)

An Alliance e2695 Separation Module coupled with a 2489 UV/Vis Detector (Waters, Milford, MA, USA) was used for HPLC. To analyze glycosylation status, CEX was performed using a ProPac WCX-10 column (Thermo Fisher Scientific) that had been equilibrated with equilibration buffer (10 mM sodium acetate, pH 4.0). Aliquots of 5 μg of mAb were filtered through 0.22-μm centrifugal filters (Merck) and applied to the column with a flow rate of 1 mL/min at 25 °C. The bound mAb was eluted with a linear gradient from 0% to 100% elution buffer (10 mM sodium acetate, pH 4.0, and 1 M sodium chloride) for 60 min. To analyze oligomeric IgGs, SEC was performed using an XBridge Protein BEH SEC 3.5-μm column (Waters) preequilibrated with Dulbecco’s PBS. Aliquots of 10 μg of sample were filtered through 0.22-μm centrifugal filters (Merck) and applied to the column. The flow rate was set to 1 mL/min and the column temperature to 30 °C during measurements. For mAb quantification, Protein A affinity chromatography was performed using a HiTrap MabSelect SuRe column (Cytiva) equilibrated with Dulbecco’s PBS. A calibration curve was prepared using 20–80 mg of purified 1C10K. Each sample was filtered through a 0.22-μm centrifugal filter (Merck) and applied to the column with a flow rate of 1 mL/min at 25 °C. The bound mAb was eluted with elution buffer (100 mM sodium citrate, pH 3.0). Peak areas were calculated from chromatograms obtained by measuring absorbance at 280 nm using Empower 3 software version 7.21 (Waters).

### 4.9. Glycan Structural Analysis

After alkylation and digestion with trypsin, *N*-linked glycans of the mAb were released by treatment with PNGase F [35] and purified using BlotGlyco (Sumitomo Bakelite, Tokyo, Japan). On-bead derivatization and labeling of the glycan were performed by the glycoblotting method [36]. The prepared samples were analyzed by MALDI-TOF MS using Ultraflex III (Bruker, Billerica, MA, USA). The glycan structure of each peak in the mass spectral data was estimated using GlycoMod [37]. The ratio of each glycan structure was calculated from the total ion intensity.

### 4.10. Luciferase-Based ADCC Assay

The luciferase-based ADCC assay was conducted as previously described [38]. Briefly, CEM.NKr-CCR5 cells with LTR-Luc (NKR24 cells) were infected with HIV-1 BaL by spinoculation in microtubes. The NKR24 target cells (1 × 10^6^) and infectious viral inoculum were centrifuged at 1200× *g* for 2 h at 25 °C. Then, the viral inoculum was removed, and the target cells were cultured in R10 medium [38]. The cells were washed three times with R10 medium and suspended in R10 medium containing 10 U/mL IL-2 without cyclosporine. In round-bottomed, tissue culture-treated polystyrene 96-well plates, 40 μL each of infected NKR24 target and N6 effector cells was added at rates of 2.5 × 10^5^ cells/mL and 2.5 × 10^6^ cells/mL, respectively. The mAbs were added to these cells in triplicate at concentrations of 0.2, 2, or 20 μg/mL, and the plates were incubated for 6 h at 37 °C in 5% CO_2_. After the reaction, a 40 μL mixture of cells and mAbs was transferred to plates for the luciferase assay and mixed with 40 μL of NeoLite Plus (PerkinElmer, Shelton, CT, USA). Luciferase activity was measured using an EnSpire multimode plate reader (PerkinElmer), and the percentage of dead HIV-1-infected cells was calculated from the decrease in relative light units (RLU). N6 effector cells and uninfected NKR24 target cells without mAbs were used as a control to define 0% RLU, and N6 effector cells and infected NKR24 target cells without mAbs were used as a control to define 100% RLU.

### 4.11. Silkworm Rearing and Cocoon Production

By crossing the 1C10K^+/+^ line with the IM71K^+/+^ line, eggs of the 1C10K^+/−^/IM71K^+/−^ line, which bore heterozygously both 1C10 mAb cDNAs and *ie1* gene, were prepared for mass rearing. The diapause of the eggs was broken by acid treatment. All rearing processes from the first-instar stage to cocoon collection were conducted in Grade C clean rooms. For rearing during the larval stages, the room temperature was controlled at 25 °C ± 2 °C with 50% ± 10% RH. First- to fifth-instar larvae were reared using paper-based rearing trays lined with plastic mesh on the bottom (Shinryo, Tokyo, Japan). An artificial diet (SilkMate PS; Nosan) was used for all larval stages. During larval stages, excluding molting periods, RH was maintained above 75% by wrapping the rearing trays in perforated plastic bags. RH was lowered to 50% ± 10% during molting by removing the trays from the bags to allow desiccation of the diet and larval fasting. This process helped to synchronize silkworm growth by aligning the timing of post-molt feeding. For cocooning and pupation, fifth-instar larvae on day 7 after molting were transferred to paper-based cocooning frames (Shinryo). Cocoons were collected from the frames after incubation for 8 days at 25 °C ± 2 °C and 25% ± 10% RH. Scaffold silk around the cocoons was removed, and pupae were taken out by cutting the upper part of the cocoons. The collected cocoon shells were stored at 4 °C until use.

### 4.12. Large-Scale 1C10K Purification

The 1C10K was extracted from cocoons by stirring in extraction buffer (20 mM sodium phosphate, pH 7.5, 150 mM sodium chloride, 4 M urea, and 0.2% *w*/*v* Triton X-100) for 90 min. For each 2 kg of cocoons, 100 L of extraction buffer was used. After the extract was collected, the cocoons were rinsed by stirring in 50 L of extraction buffer per 2 kg of cocoons for 30 min; the rinse was added to the initial extracts. The extract was clarified by filtration using a Stax large-depth filter capsule (Cytiva) connected in series with a 0.22-μm membrane filter unit (Supor EKV—Kleenpak Nova Capsules; Cytiva).

The 1C10K in the extract was purified by three-step liquid chromatography consisting of Protein A affinity chromatography, ceramic hydroxyapatite chromatography, and multimodal anion exchange chromatography. Briefly, 150 L of the clarified extract was loaded onto a Protein A affinity chromatography column packed with 2 L of MabSelect PrismA resin (Cytiva) preequilibrated with extraction buffer. The flow rate was set to 0.8 L/min. After the column was washed with extraction buffer and 40 mM sodium acetate buffer (pH 6.0), the bound mAb was eluted with 40 mM sodium acetate buffer (pH 3.5). To inactivate potential contaminating viruses, the pH of the elution was adjusted to 3.5 by adding 100 mM HCl solution, and the solution was incubated at room temperature for 2 h. Then, the pH was neutralized to 7.0 by adding 100 mM tris solution. Before subsequent ceramic hydroxyapatite chromatography, the concentrations of sodium phosphate and calcium chloride in the neutralized solution were adjusted to 10 mM and 72 μM by adding 400 mM sodium phosphate solution (pH 7.0) and 7.2 mM calcium chloride solution, respectively. The resultant 1C10K solution was loaded onto a ceramic hydroxyapatite chromatography column packed with 5 L of CHT Ceramic Hydroxyapatite XT resin (Bio-Rad Laboratories) that had been equilibrated with equilibration buffer (10 mM sodium phosphate, pH 7.0, and 72 μM calcium chloride). The flow rate was set to 1.0 L/min. After the column was washed with equilibration buffer, a surface neutralization solution composed of 25 mM tris-HCl (pH 7.8), 5 mM sodium phosphate, and 25 mM sodium chloride was added to substitute the protons on the surface of ceramic hydroxyapatite with a neutral cation. The bound mAb was eluted with elution buffer (10 mM sodium phosphate, pH 7.0, 250 mM sodium chloride, and 72 μM calcium chloride). Washing and elution were performed at a flow rate of 2.0 L/min. Finally, multimodal anion exchange chromatography was conducted using a ReadyToProcess single-use column prepacked with 1 L of Capto adhere resin (Cytiva) that had been equilibrated with 100 mM sodium citrate buffer (pH 4.5) containing 150 mM sodium chloride. The concentration of sodium citrate in the eluate from the ceramic hydroxyapatite column was adjusted to 100 mM by adding 300 mM sodium citrate buffer (pH 4.5). The solution was applied to the column at a flow rate of 0.5 L/min, followed by loading in equilibration buffer. The flow-through fraction containing unbound 1C10K was collected and neutralized to pH 7.0 by adding 1 M tris solution.

The volume of the 1C10K solution was concentrated using an Allegro CM150 auto single-use TFF system with a T-series centramate cassette (Cytiva), and the buffer was exchanged with TFF buffer (10 mM sodium acetate, pH 5.5, 50 mM sodium chloride, and 100 mM arginine). The concentration of 1C10K was adjusted to 55 mg/mL by adding TFF buffer.

### 4.13. HCP Assay

HCP levels were assayed using a Silkworm Host Cell Protein Assay Kit (Immuno-Biological Laboratories). The cocoon extract from the null silkworm Kinshu strain was used as an HCP standard.

## Figures and Tables

**Table 1:** Effects of rearing temperatures from the fourth to fifth instar stages.

		Rearing Temperature (°C)				
		20	23	25	27	30
Rearing outcomes	Rearing period (days)	17	13	11	11	11
	Cocoon formation (%)	90	95	100	100	100
	Cocoon shell weight (mg)	232 ± 56	251 ± 67	252 ± 16	244 ± 46	206 ± 45
*N*-glycosylation status	Hemi-glycosylated (%)	19.9	20.1	21.3	17.6	18.3
	Non-glycosylated (%)	1.2	1.2	1.6	1.9	1.3
*N*-glycan structures	High mannose-type (%)	66.9	66.3	70.0	70.3	69.4
	Complex-type (%)	33.1	33.7	30.0	29.7	30.6

**Table 2:** Effects of temperature and humidity in the cocooning stage.

Temperature (°C)	20 °C		25 °C			30 °C	
Humidity (%)	25%	65%	25%	45%	65%	25%	65%
Cocooning period (days)	10	10	8	8	8	8	8
Cocoon formation rate (%)	95	89	100	100	95	100	75
Cocoon shell weight (mg)	229 ± 24	222 ± 37	255 ± 21	259 ± 25	249 ± 17	198 ± 47	210 ± 42
Antibody extraction rate (%)	104	70	80	65	58	99	53

**Table 3:** Comparison of the results of three pilot-scale mass-rearing.

	Batch A	Batch B	Batch C
Number of first instar larvae	28,000	45,000	22,000
Larvae survival rate (%) 3rd instar day 1	88 ± 3	89 ± 5	93 ± 6
4th instar day 1	83 ± 2	85 ± 3	90 ± 5
5th instar day 1	82 ± 2	83 ± 4	85 ± 4
Larvae weight (g) 4th instar day 1	0.18 ± 0.02	0.19 ± 0.02	0.17 ± 0.01
5th instar day 1	0.83 ± 0.09	0.86 ± 0.10	0.80 ± 0.08
5th instar day 6	4.03 ± 0.50	4.01 ± 0.80	4.02 ± 0.52
Cocoon yield (kg)	4.6	8.0	4.0
Cocoon shell weight (mg)	222	226	221
mAb concentration in the extract (mg/mL)	0.11	0.12	0.11

**Table 4:** Summary of yields and recoveries of the purified 1C10K.

	Batch A	Batch B	Batch C
Cocoon amounts used for extraction (kg)	4.0	8.0	4.0
Antibody amount in the extract (g)	40.6	88.2	43.7
Antibody amount per 1 kg cocoons (g)	10.2	11.0	10.9
Purified antibody amount (g)	21.6	57.8	24.4
Overall recovery rate (%)	53	66	56
Purified antibody amount per 1 kg cocoons (g)	5.4	7.2	6.1

**Table 5:** Qualities of the purified 1C10K.

	Batch A	Batch B	Batch C
Antigen-binding activity (EC_50_) (mg)	0.61	0.54	0.54
*N*-glycosylation status (%) Hemi-glycosylated	16.7	16.2	16.7
Non-glycosylated	0.5	0.4	0.6
*N*-glycan structures (%) High mannose-type	71.6	70.0	72.5
Complex-type	28.4	30.1	27.5
Oligomeric IgGs (%)	0.2	0.2	0.2
HCPs (ppm)	3.9	4.6	2.9

## Data Availability

Data is contained within the article.
